# Influence of the Phenological Stage and Harvest Date on the Bioactive Compounds Content of Green Pepper Fruit

**DOI:** 10.3390/molecules26113099

**Published:** 2021-05-22

**Authors:** Alicia Dobón-Suárez, María J. Giménez, Salvador Castillo, María E. García-Pastor, Pedro J. Zapata

**Affiliations:** Department of Food Technology, EPSO, University Miguel Hernández, Ctra. Beniel km. 3.2, 03312 Alicante, Spain; alicia.dobon@goumh.umh.es (A.D.-S.); maria.gimenezt@umh.es (M.J.G.); scastillo@umh.es (S.C.); m.garciap@umh.es (M.E.G.-P.)

**Keywords:** antioxidant activity, *Capsicum annuum*, organic acids, phenolics, vitamin C

## Abstract

Green pepper fruit is often consumed before it is completely ripe. However, the influence of the phenological stage in which the green pepper is consumed as a potential influencing factor in its bioactive compounds content and antioxidant capacity remains unknown. In addition, no literature is available concerning the bioactive compounds changes in ‘Lamuyo’ green peppers along its developmental and growth cycle. For this, two different approaches have been carried out, one using twelve different phenological stages (S1 to S12), and in the other, seven different harvest dates (from 27 February to 20 April). Moreover, bioactive compounds changes during 21 days of postharvest storage at 8 °C were investigated. In this study, bioactive compounds (ascorbic acid, dehydroascorbic acid, and total phenolic content) and the total hydrophilic and lipophilic (TAA-H and TAA-L) antioxidant activity were analysed. In addition, total soluble solids, total acidity, individual sugars, and organic acids were determined. Vitamin C levels increased along the phenological stages and harvest dates due to significant increases in ascorbic and dehydroascorbic acid levels. Our results show that the total phenol content decreases as vegetables develop and subsequently increases both as ripening begins and by the last harvest date. Furthermore, TAA-H was also greater by the phenological stage S12 and the 20 April harvest date. In conclusion, the phenological stage and harvest date are key factors that significantly influence the bioactive compounds of green peppers, and those that appear by S12 and 20 April could be more beneficial to health.

## 1. Introduction

Currently, there is great interest in maintaining good health, and the intake of bioactive compounds in the diet has important health-protecting benefits related to antioxidant activities [1]. Pepper fruit (*Capsicum annuum* L.) is a vegetable of great economic importance worldwide, and it is greatly appreciated in the market for its organoleptic qualities [2], especially for its colour variability and its high levels of bioactive compounds [3]. This vegetable is high in bioactive compounds like phenols, mainly flavonoids which are a group of phenolic compounds, carotenoids, capsaicinoids, and vitamin C [3,4], and there is good correlation between its antioxidant activity and total phenolic content, and ascorbic acid content [5]. A broad number of cultivars are available in supermarkets, most of which range in colour from green to yellow, orange, red, or purple when they are completely ripe. Green peppers are often harvested before they ripen completely, and the stage of ripeness can partly account for the phytonutrient content [6]. Some authors have reported that bioactive compounds levels can vary by genotype and ripening stage in red pepper fruit [7,8]. Moreover, harvesting time is another important factor affecting the shelf-life and antioxidant capacity of peppers. Numerous biochemical and physiological changes occur at different stages of pepper development due to changes in the synthesis, transportation, and degradation of various metabolites [9], like changes in the concentration of organic acids and sugars, the synthesis and degradation of pigments and the accumulation of volatile compounds. The phytochemical changes that occur during ripening and the resulting effect on antioxidant activity are important dietary considerations that may affect the consumption of different pepper types.

The characterisation of phytochemical changes in peppers that occur during ripening is essential since these changes could affect antioxidant activities, aroma, taste, postharvest storage, and, ultimately, consumer preference [10]. Chávez-Mendoza et al. [11] have characterised the bioactive compounds and antioxidant activity of different cultivars of grafted bell pepper harvested on three different sampling dates in the same crop cycle. This study revealed differences in the content of bioactive compounds and antioxidant activity among the grafted cultivars of bell pepper and concluded that September was the best sampling date to have the highest content in bioactive compounds and strongest antioxidant capacity in these bell peppers. As far as we know, only two studies have investigated the effect of ripening stage and harvest time on the bioactive compounds content of five different coloured *Capsicum* genotypes [12] using four sweet commercial peppers [13]. However, all of these studies have focused on the changes that occur in the bioactive compounds content during the ripening process from green to red pepper fruit and highlight the benefits of consuming these peppers in a ripe and coloured stage. In addition, the results published about the influence of harvesting time on bioactive compounds changes are limited to three sampling dates, in April, May, and June, along the developmental, growth, and ripening cycle of pepper fruit [13]. In green pepper fruit like, ‘Lamuyo’, whose consumption is increasingly in demand and has an earlier and shorter-term crop cycle than other peppers (from February to April) with a staggered flowering, the effect of the harvest date on antioxidant levels remains unclear. Furthermore, the influence of the phenological stage in which the green pepper is consumed as a potential influencing factor in its bioactive compounds content and antioxidant capacity remains also unknown. For this reason, two different approaches have been carried out in the present work. In one, we have studied twelve different phenological stages of green pepper fruit, and in the other, we have analysed green pepper fruit with the same phenological stage harvested at seven different sampling dates throughout the winter and spring periods of the same growth cycle. The aim of this work was therefore to elucidate the influence of the phenological stage and harvest date on the bioactive compounds content of green pepper fruit in order to fill this knowledge gap. In addition, we have also looked at the postharvest behaviour of bioactive compounds in order to study how the functional and nutritive parameters behave during 21 days of storage at 8 °C.

## 2. Results and Discussion

### 2.1. Effect of the Phenological Stage and Harvest Date on Ascorbic Acid (AA) and Dehydroascorbic Acid (DHA) Content

**Figure 1:**
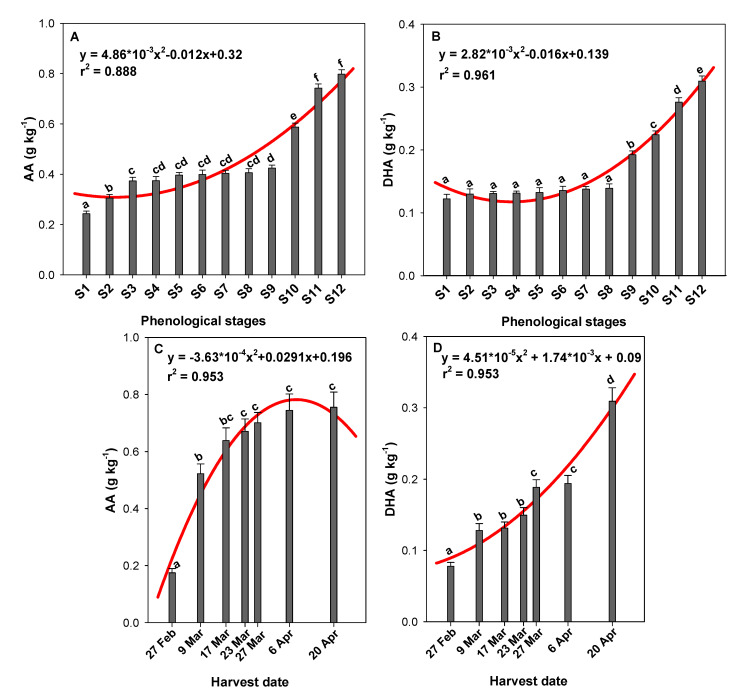
Influence of phenological stages (**A**,**B**) and harvest date (**C**,**D**) on ascorbic acid (AA) and dehydroascorbic acid (DHA) content (g kg^−1^) in green pepper fruit. Lowercase letters show significant differences (*p* < 0.05) among phenological stages or harvest dates. A quadratic model regression and its coefficients are shown in each graph.

Fresh peppers present high levels of vitamin C reaching more than three times the recommended dietary allowance (RDA) [14]. Vitamin C is one of the most important water-soluble vitamins for human health, known for its high antioxidant activity. It has two active forms: ascorbic acid (AA), which is the main one, and the oxidized form dehydroascorbic acid (DHA), which also has biological activity and is easily transformed to ascorbic acid in the human body, making it interesting to measure [13]. The vitamin C content of foods is usually considered to be the sum of the AA and DHA [15]. Figure 1A,B show the AA and DHA content, respectively, found in the green pepper fruits harvested during the twelve phenological stages of this study. Both parameters showed an increasing trend defined by a quadratic model regression (AA: y = 4.86 × 10^−3^x^2^ − 0.012x + 0.32, r^2^ = 0.888; DHA: y = 2.82 × 10^−3^x^2^ − 0.016x + 0.139, r^2^ = 0.961). The statistical analysis revealed significant differences (*p* < 0.05) for both ascorbic acid forms among the studied phenological stages. Specifically, AA increased significantly (1.75-fold) from S1 to S9, and then a sudden 1.88-fold increase was observed between S9 and S12 (Figure 1A). On the other hand, DHA levels did not show significant differences (*p* ≥ 0.05) until the green pepper fruit reached the S8 phenological stage. Between this stage and the last phenological stage, a significant 1.61-fold increase was observed (Figure 1B). The total vitamin C content, as the sum of both forms, was therefore significantly influenced by the phenological stage of the green pepper fruit, and a 3-fold increase in total content was observed between the first and last stage.

With respect to the influence of harvest date on AA and DHA content, the results are shown in Figure 1C,D, respectively. An increasing trend was observed again for both functional parameters with two new quadratic model regressions (AA: y = −3.63 × 10^−4^x^2^ + 0.029x − 0.196, r^2^ = 0.953; DHA: y = 4.51 × 10^−5^x^2^ + 1.74 × 10^−3^x + 0.09, r^2^ = 0.953). The green peppers harvested on the first harvest date, 27 February, showed significantly lower average levels of AA (0.18 ± 0.02 g kg^−1^) and DHA (0.08 ± 0.01 g kg^−1^) than those harvested on the later harvest dates. By the second harvest date, 9 March, the average AA and DHA content had significantly increased, by 2.99- and 1.65-fold, respectively (Figure 1C,D, respectively). From this point, the AA content increased 1.28-fold in the green peppers until 23 March, when the levels remained constant without significant differences until the last harvest date (Figure 1C). The DHA content, on the other hand, did not significantly increase between 9 March and 23 March. This oxidant form of ascorbic acid started to increase again by 27 March, when it showed a 1.26-fold increase with respect to the 23 March harvest date. In addition, DHA significantly increased by the last harvest date of the growth cycle, when it reached average levels of 0.31 ± 0.02 g kg^−1^ (Figure 1D).

Some authors have found differences of 30% in ascorbic acid levels between red and green peppers [7]. Specifically, Zhuang et al. [16] have reported that the vitamin C content in nine fresh peppers ranged from 0.93 g kg^−1^ FW for Creasing Pepper (green pepper) to 3.93 g kg^−1^ FW for Long-Point Pepper (red pepper), depicting a 4-fold variation between cultivars. Our total vitamin C content results (~0.25–1.11 g kg^−1^ FW) are within the ranges found in other studies for green peppers (0.12–1.80 g kg^−1^ FW) [17]. Peppers are a good source of vitamin C. Per 100 g, fresh peppers exceed the recommended daily allowance (RDA) of 60 mg [18], especially peppers harvested in S9–12 (Figure 1A,B) or those harvested from 9 March to 20 April (Figure 1C,D). These results highlight the importance of the phenological stage and harvest date on vitamin C content in green pepper fruit. Navarro et al. [19] showed that the ascorbic acid content in peppers also depends on the ripening stage. Accordingly, Ghasemnezhad et al. [12] have reported that fully developed bell pepper fruit just before the onset of colour change had more ascorbic acid than whole-coloured fruit. In a previous work, vitamin C tended to increase at the onset of ripening, but then decreased gradually with the advanced ripening most probably due to its antioxidant role, which increases with the increasing respiration rate in the climacteric fruit [20]. In green pepper fruit, which is harvested before full ripening and therefore does not undergo colour changes, the ascorbic acid content increases in the most advanced phenological stages, when the pepper fruit is fully developed (Figure 1A,B), confirming the last hypothesis. In addition, this content increased from the end of February until the end of April (Figure 1C,D). Martí et al. [13] have observed that ripe ‘Almuden’ and ‘Cabezo’ sweet pepper cultivars show an increase in vitamin C content in May. Accordingly, we found that ‘Lamuyo’ green pepper fruit, which is characterized by a shorter-term crop cycle than other cultivars, shows an increase in total vitamin C content along its developmental and growth cycle. A wide range of ascorbic acid levels has been reported in a number of pepper cultivars, indicating that the differences are related to cultivar, genetics, ripening stages, and agro-climatic conditions [14,21]. Mozafar [22] has suggested that the higher level of ascorbic acid in the ripening stage is due to the light intensity and glucose levels, which are the precursors of ascorbic acid. The higher ascorbic acid levels in ‘Lamuyo’ pepper fruit (Figure 1A–D) observed on 20 April could therefore be related to the significantly higher endogenous glucose levels found at this harvest date (Figure 5A).

Under oxidative conditions, AA is easily converted through a free radical intermediate to dehydroascorbic acid (DHA) in a reversible process which, in part, may explain the antioxidant effect attributed to AA. DHA is quite unstable and, under continued oxidative conditions, is further degraded to 2,3-diketogulonic acid, which is not biologically active as vitamin C. We measured both ascorbic acid forms, and the results show that the latest phenological stages and harvest dates presented significantly higher AA and DHA levels than the other stages and dates (Figure 1A–D). Martí et al. [13] have also measured the oxidised form of ascorbate (DHA) in sweet peppers and found that different factors are operating in the different cultivars during ripening, affecting the ascorbate oxidation. A decrease in ascorbic acid usually coincides with ripening and with an increase in ascorbate peroxidase activity [23]. This enzyme catalyses the oxidation of ascorbate [24], influencing the redox state of ascorbate. Despite this effect, the ascorbic acid content was not found to decrease during ripening in ‘Lamuyo’ green pepper fruit, while a significant increase in DHA content was observed by 20 April (Figure 1D), when higher temperatures and solar radiation influenced oxidation according to other studies [13]. Finally, variability in vitamin C could be influenced by different factors at harvest. Some authors have reported that the ripening stage, cultivar or growing conditions of a particular sample of a given plant could result in different levels of antioxidant compounds, which could affect the chemical stability of AA and DHA, and/or the enzyme activity [25]. Our results could contribute two additional key factors that can also impact antioxidant compounds levels: the phenological stage and the harvest date along the growth cycle of the green pepper fruit.

### 2.2. Effect of the Phenological Stage and Harvest Date on Total Phenol Content and the Total Hydrophilic (TAA-H) and Lipophilic (TAA-L) Antioxidant Activity

**Figure 2:**
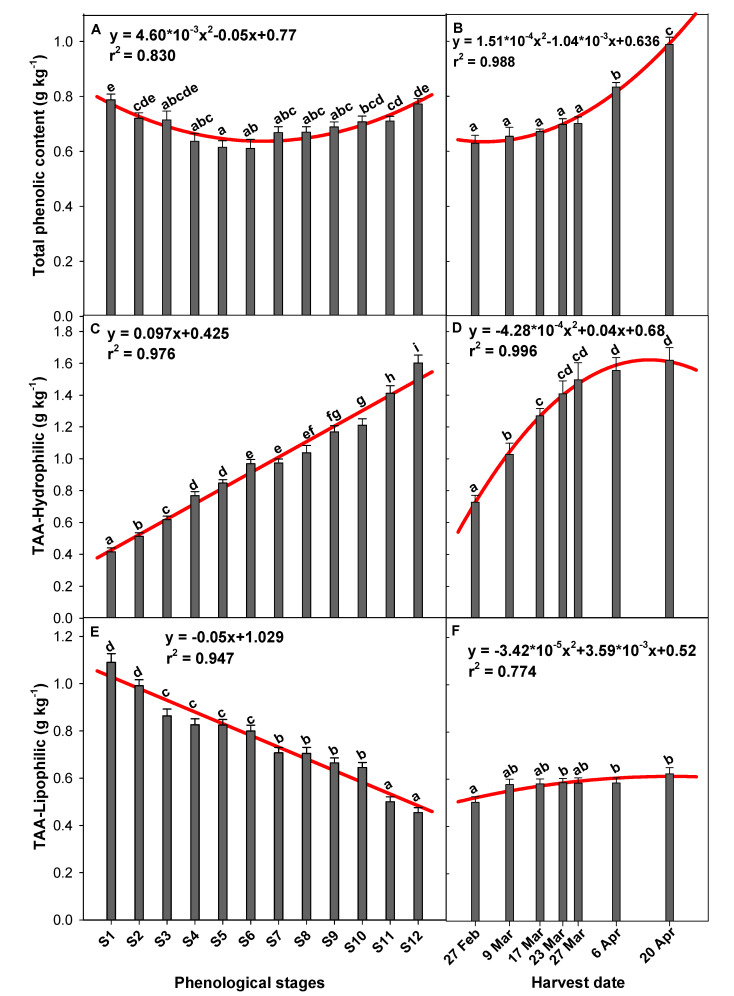
Influence of phenological stages (**A**,**C**,**D**) and harvest date (**B**,**D**,**F**) on total phenolic content (**A**,**B**), and the TAA-Hydrophilic (**C**,**D**) and Lipophilic (**E**,**F**) (g kg^−1^) levels of green pepper fruit. Lowercase letters show significant differences (*p* < 0.05) among phenological stages or harvest dates. A quadratic model regression and its coefficients are shown in each graph.

Compounds that contribute to the total antioxidant activity (TAA) in pepper fruits are numerous and include ascorbic acid and phenolic compounds, mainly flavonoids which are a group of phenolics. These two compounds are thus important in assessing their quality. Specifically, the main individual phenolic compounds in green pepper fruit are caffeic acid derivative and trans-*p*-coumaroyl-β-D-glucopyranoside such as hydroxycinnamic acids, and quercetin-3-*O*-rhamnoside and luteolin 7-*O*-(2-apiosyl-6-malonyl)-glucoside which are flavonoids [21]. All these bioactive compounds are determinant on the organoleptic characteristics (colour, appearance, flavour, and taste) and functional quality of fruits and vegetables, as well as a parameter involved in enzymatic browning and other reactions which could occur during fruit processing. The results obtained for total phenolic compounds in ‘Lamuyo’ pepper fruits at the different phenological stages showed a quadratic regression (Figure 2A; y = 4.60 × 10^−3^x^2^ − 0.05x + 0.77, r^2^ = 0.83). Peppers from earlier (S1 and S2) and later (S10–12) phenological stages showed significantly higher total phenol levels (between 1.15 and 1.11-fold higher, respectively) than the intermediate stages (S3 to S9). Harvest date also affected the total phenol content, which reached values of 0.83 ± 0.02 and 0.99 ± 0.03 g kg^−1^ by the two last harvest dates (6 and 20 April), respectively, but no significant differences were observed before these sampling dates. This increasing trend seen in the effect of harvest time on total phenol content is represented by a quadratic regression equation (y = 1.51 × 10^−4^x^2^ − 1.04 × 10^−3^x + 0.636, r^2^ = 0.988) in Figure 2B.

On the other hand, a significant increasing trend of TAA-Hydrophilic was defined for the phenological stage in a linear model (Figure 2C; y = 0.097x + 0.425, r^2^ = 0.976) and for harvest date in exponential form (Figure 2D; y = −4.28 × 10^−4^x^2^ + 0.04x + 0.68, r^2^ = 0.996), respectively. Specifically, the green peppers harvested at S12 and on the last two harvest dates (6 and 20 April) showed the highest TAA-Hydrophilic values (~1.59 g kg^−1^; Figure 2C,D). Nevertheless, TAA-Lipophilic values showed a drastic decrease as the phenological stages advanced (Figure 2E), resulting in 2.40-fold variations. This descent was drawn with a quadratic regression; y = −0.05x + 1.029 with values of r^2^ = 0.947. Contradictorily, the harvest date factor showed a 1.24-fold increase in TAA-Lipophilic values along the developmental and growth cycle in ‘Lamuyo’ peppers (Figure 2F; y = −3.42 × 10^−5^x^2^ + 3.59 × 10^−3^x + 0.52, r^2^ = 0.774).

Phenolic compounds are secondary plant metabolites that play an essential role in antioxidant activity. The total phenol content of ‘Lamuyo’ pepper (~0.60–1.00 g kg^−1^; Figure 2A,B) was lower than that reported in other green-stage pepper cultivars (~2.10 to 5.58 g kg^−1^) [10]. However, our total phenol content was accordance with the results observed in the Sweet/Robusto cultivar (green bell pepper fruit), averaging 0.70 g kg^-1^, by Chávez-Mendoza et al. [11]. This is the first report in which the effect of the phenological stage on total phenol content has been analysed, and the results show that this content decreases as the vegetable develops and subsequently increases as ripening begins but prior to full ripening (Figure 2A). The highest total phenol content values were thus observed at the S1 and S12 phenological stages. A similar effect or downward trend was observed in lemon fruit during growth and ripening on trees from 11 September through 13 December [26].

The increase in total phenolic content during the last phenological stages could be related with earlier reports suggesting that fruit and vegetable ripening is associated with a significant accumulation of total phenolic content [27]. Phenolic compounds are produced by the phenylpropanoid pathway during ripening and contribute to fruit pigmentation and the disease resistance response found in many fleshy fruits [28]. In general, the mature red stage displays a higher content of total phenolics than the green stage in pepper fruits [16]. The type of green pepper fruit in this study, however, is harvested and consumed before reaching the colour changes associated with the ripening process. According to Ghasemnezhad et al. [12], a high concentration of quercetin in green pepper fruit may be connected with the function of protecting the photosynthetic apparatus. It was shown that flavonoids, which strongly absorb radiation within the range 280–315 nm, that is UVB, could act as filters of UV radiation, in this way protecting photosynthesizing cells-that are situated deeper-against damage.

The significant increase noticed on the last two harvest dates (6 and 20 April; Figure 2B) was also observed in previous studies, which found that harvest date is an important factor that influences the total phenol content in pepper fruit [11,12,13]. However, the total phenol increase in the present work along the different harvest dates could be related to the increase in temperatures during the month of April. Accordingly, it has been reported that the season in which pepper fruits develop influences changes in phenolic compounds content. Nevertheless, the developmental conditions of the fruits, as well as the cultivar analysed, are decisive factors influencing antioxidants like phenolic compounds in fruits and vegetables [14].

Ultimately, the antioxidant capacity is given by compounds present on hydrophilic and lipophilic fractions (Figure 2C–F). Lipophilic compounds are mainly chlorophylls, carotenoids, and vitamin E, while the hydrophilic ones are vitamin C, glutathione (GSH), and phenols, mainly flavonoids. Both antioxidant activity fractions are related to potential health functionality against various chronic non-communicable diseases [1]. Some authors have reported that the TAA of pepper cultivars increases significantly with ripening [12,14,19]. Our TAA-Hydrophilic and TAA-Lipophilic results along the phenological stages show for the first time that the contribution of both fractions is inversely proportional as the pepper develops. Green pepper fruits harvested at S1 showed higher TAA-Lipophilic values than those harvested at S12, but the opposite occurred with the antioxidant activity provided by hydrophilic compounds (Figure 2C,E). The difference in the antioxidant activities reflects the nature and level of the antioxidant compounds found in green pepper fruit. The increase in TAA-Hydrophilic levels along the phenological stage could be related to increases in both ascorbic acid content, in AA and DHA forms (Figure 1A,B), and phenolic compounds (Figure 2A). However, the increasing TAA-Hydrophilic levels seem to be more related to the observed increase in vitamin C than the total phenolic content in the last phenological stages (Figure 2C). On the other hand, the drastic decrease in TAA-Lipophilic levels seem to be mediated by the loss of some lipophilic compounds along the phenological stages studied (Figure 2E). Chlorophylls are the main compounds that change during pepper development on the plant, and lipophilic-nature pigments are responsible for the characteristic green colour of each cultivar. As can be seen in Figure S1, the green colour of ‘Lamuyo’ pepper fruit ranged from light green in S1 to a deep green in S12. Currently, a new and promising set of assays showing the health-promoting activities of chlorophylls has promoted the development of studies dealing with their in vivo antioxidant actions [29]. Enhanced TAA at the fully developed stage of green pepper fruit, mainly provided by the TAA-Hydrophilic fraction, reflects the nutritional and functional importance of consuming the pepper fruits at this stage.

Finally, both TAA-Hydrophilic and TAA-Lipophilic levels increased as the harvest date progressed (Figure 2D,F). These results agree with those reported by Chávez-Mendoza et al. [11] and Martí et al. [13]. Again, results point to the importance of environmental factors like temperature, radiation, and humidity in fruits development. Higher temperatures and solar radiation seem to be good for fruits in order to increase the levels of both TAA fractions.

### 2.3. Evolution of Functional Parameters during Postharvest Storage

**Figure 3:**
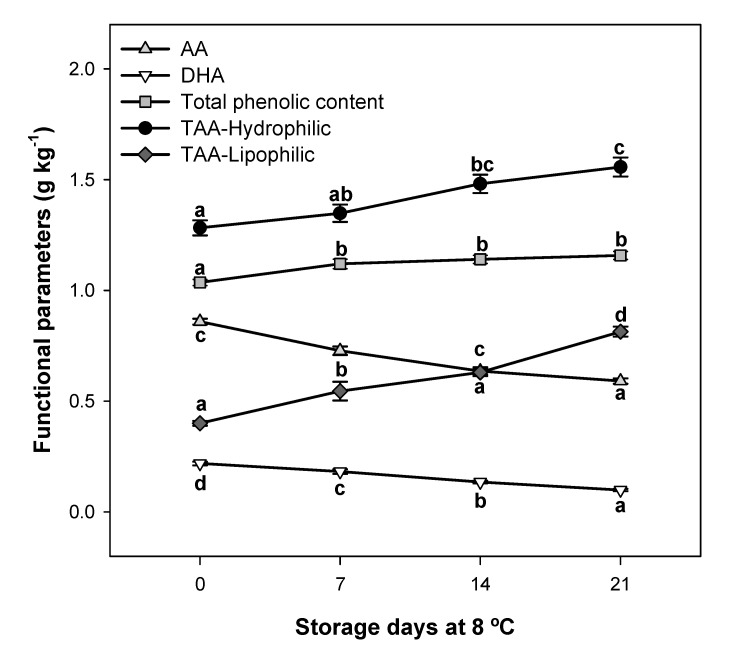
Evolution of functional parameters (g kg^−1^); ascorbic acid (AA), dehydroascorbic acid (DHA), total phenolic content, and TAA-Hydrophilic and TAA-Lipophilic levels during 21 days of postharvest storage at 8 °C. Lowercase letters show significant differences (*p* < 0.05) among storage days at 8 °C.

The bioactive compounds behaviour of pepper fruit at 8 °C (non-chilling temperature) during postharvest storage is shown in Figure 3. The AA and DHA content significantly decreased (by 31 and 55%, respectively) by 21 days of storage. Nevertheless, the total phenolic content and TAA-Hydrophilic and TAA-Lipophilic levels were significantly higher (1.12, 1.21, and 2.03-fold, respectively) at the end of postharvest storage. It has been described that vitamin C levels in fruits depend on storage conditions among other factors, including the cultivar, production practices or ripening stage [14,21,25,30]. Vitamin C loss in the green peppers studied is in accordance with that observed by Barzegar et al. [31] and could be due to chemical (non-enzymatic oxidation), and/or enzymatic processes. It is worth mentioning the effect of the enzymes on L-AA stability, especially in fresh produce, in which enzymatic activity may be an important contributor to L-AA degradation. During weight loss, ascorbic acid can be rapidly lost as a result of oxidation [25]. In addition, substantial vitamin C degradation could occur due to the storage time, temperature or exposure to light [25,32,33].

Our results show that total phenolic content and TAA-Hydrophilic and TAA-Lipophilic levels increased in ‘Lamuyo’ green pepper fruit up to 20 days of storage at 8 °C (Figure 3). Similar results were reported by Barzegar et al. [31] and Barbagallo et al. [34], who found that that the total phenolic content and DPPH scavenging activity in control green peppers increased up to day 20 and the 3rd week of storage, respectively, and then decreased. Phenolic compounds accumulated in pepper fruit are affected by storage condition. Raffo et al. [35] demonstrated that sweet peppers stored at 8 °C accumulated hydroxycinnamic acid derivatives, whereas at 4 °C phenolics accumulation appeared to be partially inhibited. Our results about the increase of total phenolic content and total antioxidant activity during 21 days of storage at 8 °C could be related with an increase of hydroxycinnamic acid derivatives.

### 2.4. The Effect of the Phenological Stage, Harvest Date, and Postharvest Storage on Total Soluble Solids (TSS) and Total Acidity (TA)

**Figure 4:**
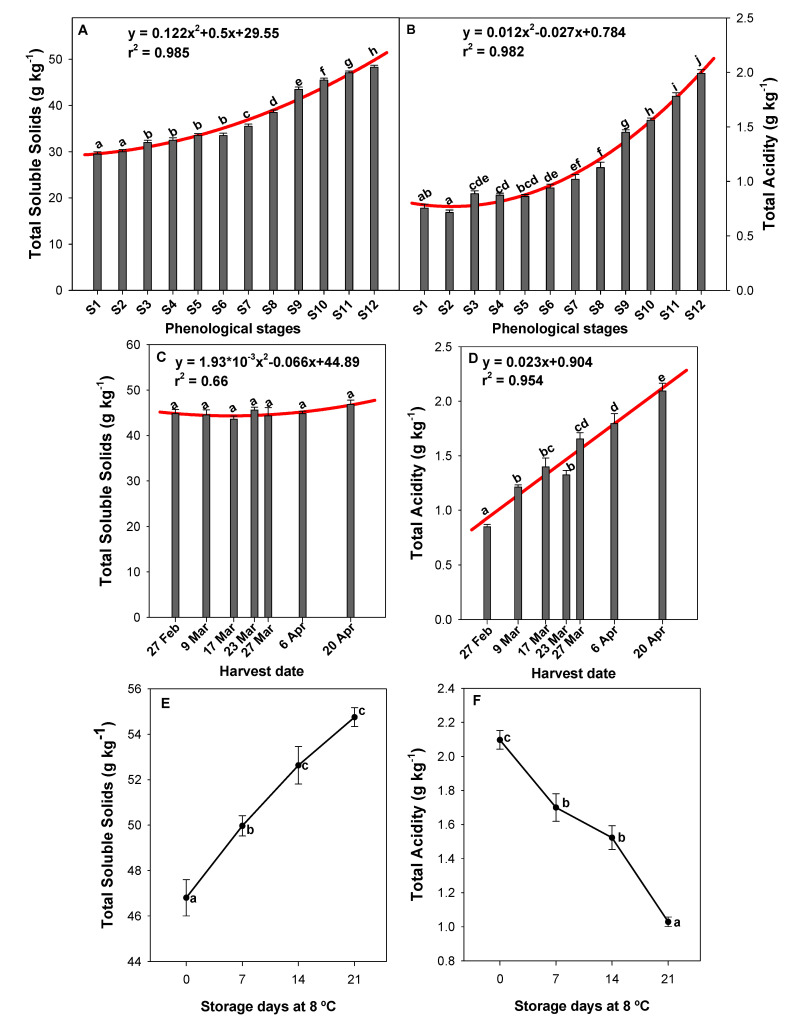
Influence of phenological stages (**A**,**B**), harvest date (**C**,**D**), and postharvest storage (**E**,**F**) on total soluble solids (TSS) (**A**,**C**,**E**) and total acidity (TA) (**B**,**D**,**F**) (g kg^−1^) in green pepper fruit. Lowercase letters show significant differences (*p* < 0.05) among phenological stages, harvest dates, or storage days at 8 °C. A quadratic model regression and its coefficients are shown in some graphs.

The phenological stage influenced the TSS and TA content (Figure 4A,B). Both parameters significantly increased at S12, reaching values of 48.30 ± 0.41 and 1.99 ± 0.04 g kg^−1^, respectively. The trend was described by a quadratic model correlation for TSS (y = 0.122x^2^ + 0.5x + 29.55, r^2^ = 0.985) and TA (y = 0.012x^2^ − 0.027x + 0.784, r^2^ = 0.982). Furthermore, harvest date was also highly correlated with the TA content (y = 0.023x + 0.904, r^2^ = 0.954; Figure 4D), but no significant differences were observed in TSS among the studied harvest dates (Figure 4C). Therefore, green pepper fruits harvested on 20 April showed 2.46-fold higher total acidity levels than peppers harvested on 27 February (Figure 4D). TSS and TA were also affected by postharvest storage, showing an opposite trend (Figure 4E,F). The TSS content in green peppers had significantly increased, by 15%, by 21 days of storage at 8 °C (Figure 4E), while the TA had significantly decreased, by 51%, by the end of the storage period (Figure 4F).

It has been reported that TSS content increases with fruit ripening as a result of greater degradation of the polysaccharides and accumulation of sugars [36], confirming that our TSS values at S12 were the highest (Figure 4A). However, no significant differences were observed for TSS along the harvest dates (Figure 4C). This is because the ‘Lamuyo’ pepper fruits were harvested on different dates according to commercial criteria for TSS (~45.00 g kg^−1^).

**Figure 5:**
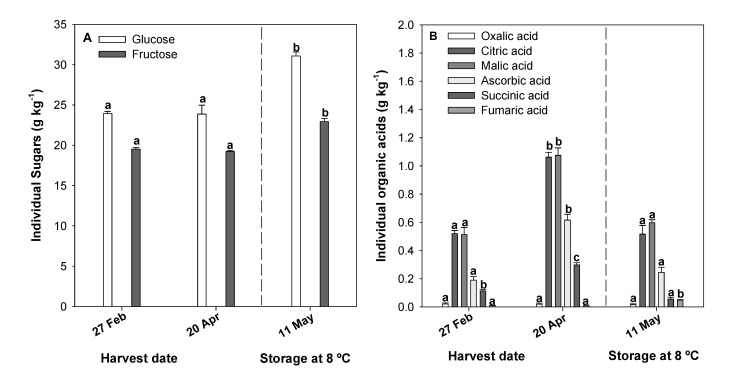
Influence of harvest date (27 February and 20 April) and postharvest storage (at harvest; 20 April, and at 21 storage days; 11 May) on individual sugar (**A**) and organic acid (**B**) levels (g kg^−1^) in green pepper fruit. Lowercase letters show significant differences (*p* < 0.05) among harvest dates and storage days at 8 °C.

The main organic acids contributing to pepper acidity were citric and malic acid (Figure 5B), and TA increased with fruit ripening (Figure 4B), as previously reported by Serrano et al. [37]. The increases in TA along the different harvest dates (Figure 4D) could be related to an increase in the average temperature in April, as has been reported by Hernández-López et al. [38]. This is because metabolic processes occur more slowly in low temperature conditions, while an increase in temperature causes an opposite effect. Finally, the increase in TSS content could potentially be due to pepper fruit weight loss and the conversion of organic acids to sugars [39]. Organic acid losses lead to significant decreases in TA values from harvest up until 21 days of storage (Figure 4F), according to Barzegar et al. [31].

### 2.5. The Effect of Harvest Date and Postharvest Storage on Individual Sugars and Organic Acid Content

Changes in TSS an TA are based on changes in individual sugars and organic acid content (Figure 5A,B) along the harvest dates (27 February to 20 April) and during postharvest storage (from 20 April to 11 May). No significant differences were observed for the individual sugars, glucose and fructose, between the first harvest date, 27 February, and the last one, 20 April (Figure 5A). The ‘Lamuyo’ pepper showed 55% glucose and 45% fructose at the first harvest date, and no changes in the sugar profile were found by the last harvest date. Nevertheless, a significant increase in citric, malic, and ascorbic acid (51%, 53%, and 69%, respectively) was observed by 20 April (Figure 5B).

The organic acid profile of ‘Lamuyo’ green peppers was as follows: fumaric acid (0.67%), oxalic acid (1.47%), succinic acid (8.09%), ascorbic acid (13.98%), malic acid (37.53%), and citric acid (38.26%). This is similar to the profile reported by Serrano et al. [37]. However, these researchers reported that citric acid was the main organic acid contributing to pepper acidity in the ‘Herminio’ red cultivar. Our results showed that the proportion of malic and citric acid was similar in the green peppers of this cultivar (Figure 5B). Finally, the glucose and fructose levels were significantly higher (1.22 and 1.15-fold, respectively) after 21 days of storage at 8 °C (Figure 5A). By the end of the storage period, the levels of citric, malic, ascorbic, and succinic acids had significantly decreased, by 51%, 44%, 60%, and 82%, respectively; fumaric acid, on the other hand, increased by 83% (Figure 5B). The organic acid profile thus changed during 21 days of postharvest storage at 8 °C.

No differences in glucose and fructose content were observed along the harvest dates, because all pepper fruits were harvested with the same commercial criteria (Figure 5A). Nevertheless, the increase in both individual sugars during postharvest storage could be related to the ripening process that continuously occurs under storage at 8 °C (Figure 5A), according to Mashabela et al. [36]. With respect to organic acid profile (Figure 5B), Barzegar et al. [31] have also reported changes in the citric, malic, and ascorbic acid content of sweet pepper fruits. Concentrations of these acids are known to diminish during ripening. Our organic acid results are in accordance with previous reports showing that overall acidity increases after harvest and then decreases in storage [40]. Medlicott and Thompson [41] also reported reduced acidity levels with prolonged storage due to the fact that the predominant malic acid diminishes in the fruit as the ripening process advances. Carbohydrate and acid metabolism are therefore closely connected during the postharvest ripening period [42]. Finally, these compositional differences on TSS, TA, individual sugars, and organic acid content could determine sensory differences of perceived sweetness as well as could be related with the consumers’ preferences. Sweet pepper taste is largely determined by the sugar-to-acid ratio and, in general, these two components can vary independently altering taste attributes of fruit and vegetables [35].

## 3. Materials and Methods

### 3.1. Green Pepper Fruit Cultivar and Growing Conditions

‘Lamuyo’ pepper plants (*C. annuum* L.), of the ‘Herminio’ cultivar, were grown under plastic-roofed greenhouses (Hortalizas Sanper S.L., El Raal, Murcia, Spain). The experiment was carried out during the winter-spring season (February–April 2020). According to the usual crop programme designed by the company for the early cycle of this type of pepper, automatic drip irrigation and optimal nutrient levels were applied and rockwood was used as the soil substrate. The soil texture was sandy loam with a pH of 7.50. Meteorological data were collected from a station close to the experimental greenhouses (38°2′2.64″ North, 1°1′18.9″ West). The mean long-term climate data during the growing season (2020) is shown in Table 1. The green pepper fruits were harvested on 10 April at different phenological stages in the developmental and growth cycle; we also harvested fruits on seven different harvest dates, ranging from the end of February to the end of April. Pepper fruit harvested on different harvest dates were from the same phenological stage (S12). For this reason, the importance to study, on the one hand, the influence of different phenological stages on the bioactive compounds content and, on the other hand, the influence of different harvest dates on this content in peppers with the same phenological traits. We used three replicates of 30 plants (*n* = 90 plants) for these two approaches. The pepper fruits were harvested and classified at 12 different phenological stages, from S1 until S12, along the crop cycle. The biometrical characteristics of the stages studied are described in Figure S1. Specifically, ten green pepper fruits, one fruit per plant (*n* = 10 plants), were analysed for each replicate and phenological stage (*n* = 30 green fruit per stage). Previous to the analytical determinations, the green peppers harvested from each replicate were weighed (g) and measured in length and diameter (mm), and results were expressed as the mean ± SE (Figure S1). For the harvest date study, green-pepper fruits were harvested from 10 different plants per replicate (*n* = 30 pepper plants) along the short-term crop cycle of ‘Lamuyo’ type. According to a staggered production, 10 green peppers, one fruit per plant from each replicate (*n* = 30 green peppers), were analysed in each harvest date (*n* = 210 pepper fruit per crop cycle) and phenological stage (*n* = 30 green pepper fruit per stage). The harvest dates studied were 27 February, 9 March, 17 March, 23 March, 27 March, 6 April, and 20 April. The equidistance among harvest dates was stablished according to commercial criteria of harvesting in green pepper fruit established by the company. All green pepper fruits were cut to remove the peduncle and the seeds and then frozen in liquid N_2_, and maintained at −80 °C until analysis.

### 3.2. Experimental Postharvest Storage Design

At the end of the harvest period, 20 April, 40 green pepper fruits that were homogeneous in colour and size were selected from another 10 different pepper plants for each replicate (*n* = 120 green peppers). The pepper fruits were immediately transferred to the laboratory and randomly divided into four batches of 30 green peppers each. One batch was analysed after harvest and the other ones stored at 8 ± 1 °C and 85% Relative Humidity (RH) for 21 days (until 11 May). At 7-day intervals, one group was taken at random and subjected to the following analyses.

### 3.3. Ascorbic Acid (AA) and Dehydroascorbic Acid (DHA)

Ascorbic (AA) and dehydroascorbic (DHA) acids were measured according to the method of Peña-Estévez et al. [43]. Briefly, 5 g of frozen green pepper fruit was homogenised with 5 mL of a methanol: water (5:95) solution containing 0.1 mM citric acid, 0.05 mM ethylenediamine tetraacetic acid disodium salt, and 4 mM NaF for 30 s on an Ultraturrax (T18 basic, IKA, Berlin, Germany). Then, the extract was filtered through a four-layer cheesecloth and the pH was adjusted to 2.35–2.40 with 2 N HCl; it was then centrifuged at 10,000 *g* for 15 min at 4 °C. The supernatant was purified through a methanol-activated C_18_ cartridge (Sep-Pak cartridges C18, Waters, Dublin, Ireland) and filtered through a 0.45 μm PFTE filter. For DHA derivatization, 750 μL of extract were mixed with 250 μL of 7.7 M 1,2-phenylenediamine in an HPLC amber vial. The mixture was allowed to react for 37 min and then 20 μL were injected onto a Luna (250 mm × 4.6 mm, 5 μm particle size) C18 column (Phenomenex, Macclesfield, UK) with a C18 security guard (4.0 mm × 3.0 mm) cartridge system (Phenomenex) using an HPLC system (1200 Infinity series, Agilent Technologies, Waldbronn, Germany). The mobile phase was 50 mM KH_2_PO_4_ containing 5 mM hexadecyl trimethylammonium bromide and 5% methanol (pH 4.59) with an isocratic flow of 1 mL min^−1^. Absorbance was recorded at 261 nm for AA (Rt = 9.4 min) and at 348 nm for DHA (Rt = 4.5 min), and both values were quantified by comparison with AA and DHA standard areas (Sigma-Aldrich, Darmstadt, Germany). Vitamin C was defined as the sum of both AA and DHA content. The results (mean ± SE) were expressed as g kg^−1^ fresh weight (FW).

### 3.4. Total Phenolic Content and Total Hydrophilic (H-TAA) and Lipophilic (L-TAA) Antioxidant Activity

To measure the total phenolic content and total antioxidant activity (TAA), 5 g of frozen green pepper fruit were homogenised in 10 mL of 50 mM phosphate buffer pH = 7.8 and 5 mL of ethyl acetate. The homogenate was centrifuged at 10,000 *g* for 15 min at 4 °C and the upper and lower fractions were used to quantify total lipophilic (L-TAA) and hydrophilic (H-TAA) antioxidant activity, respectively. In addition, the total phenol content was quantified in duplicate on the lower fraction for each extract using the Folin-Ciocalteu reagent as previously described [44]. The results were expressed as g gallic acid equivalent (GAE) kg^-1^ and are the mean ± SE of three replicates. H-TAA and L-TAA were determined in duplicate in each extract as previously described, also by Sayyari et al. [44]. A reaction mixture containing 2-2′-azino-bis-(3-ethylbenzothiazoline-6-sulfonic acid) diammonium salt (ABTS), horseradish peroxidase enzyme, and its oxidant substrate (hydrogen peroxide) was performed to monitor at 730 nm the ABTS^+^ radicals generated. The decrease in absorbance after adding the green pepper extract was proportional to the TAA of the sample calculated using a calibration curve made with Trolox [(*R*)-(+)-6-hydroxy- 2, 5, 7, 8-tetramethyl-croman-2-carboxylic acid] (0–20 nmol) from Sigma Aldrich (Madrid, Spain). The results are expressed as g of Trolox Equivalent (TE) kg^−1^ FW and are the mean ± SE of three replicates.

### 3.5. Total Soluble Solids (TSS) and Total Acidity (TA)

Ten frozen pepper samples of each replicate were combined to obtain a homogeneous sample of juice for each replicate. Total soluble solids (TSS) were measured in duplicate in the same juice using a digital refractometer (Atago PR-101, Atago Co., Ltd., Tokyo, Japan) at 20 °C and expressed as g kg^−1^ FW. Titratable acidity (TA) was determined in duplicate in each sample using 1 mL of diluted juice (in 25 mL distilled H_2_O) obtained from 50 g of pepper fruit, which was automatically titrated (785 DMP Titrino, Metrohm, Burladingen, Germany) with 0.1 N NaOH up to pH 8.10; the results were expressed as g malic acid equivalent kg^−1^ FW.

### 3.6. Individual Sugars and Organic Acids

For sugar and organic acid quantification, extraction was performed according to the protocol described by García-Pastor et al. [45]. The supernatant was filtered through a 0.45 μm Millipore filter and injected into a high-performance liquid chromatography (HPLC) system (Hewlett-Packard HPLC series 1100, Waldbronn, Germany). The elution system consisted of 0.1% phosphoric acid running isocratically with a flow rate of 0.5 mL min^−1^ through a Supelco column (Supelcogel C-610H, 30 cm 7.8 mm, Supelco, Bellefonte, PA, USA). Sugars were detected by a refractive index detector and organic acids by absorbance at 210 nm. The results were expressed as g kg^−1^ FW and are the mean ± SE of three replicates. A standard curve of pure sugars and organic acids purchased from Sigma (Poole, UK) was used to quantify these compounds.

### 3.7. Statistical Analysis

The analysis was carried out in three replicates for all analytical determinations. Results are expressed as mean ± SE. Data were subjected to analysis of variance (ANOVA). The sources of variation were the phenological stages, harvest dates or storage time. Mean comparisons were performed using Tukey’s HSD test to determine whether the differences among the phenological stages, harvest dates or storage time were significant at *p* < 0.05. All analyses were performed using the SPSS software package v.17.0 for Windows (SPSS, 2001, IBM Corporation, Armonk, NY, USA).

## 4. Conclusions

In conclusion, phenological stages and harvest dates are two key factors that significantly influence the bioactive compounds content and the antioxidant activity of ‘Lamuyo’ green pepper fruit. Firstly, green peppers harvested in S12 and on 20 April showed the highest levels of antioxidant compounds, mainly ascorbic acid, dehydroascorbic acid, and total phenolic content. Secondly, these peppers showed the highest total acidity due to the significant increase in citric, malic, ascorbic, and succinic acids at these two points. Thirdly, some of these bioactive compounds and organic acids significantly decreased during 21 days of postharvest storage at 8 °C. Therefore, it is advisable to harvest the green pepper fruits at the most advanced phenological stage (S12) and on the latest harvest dates, in April, in order to achieve maximum health benefits in terms of functional traits.

## Figures and Tables

**Table 1:** Meteorological data provided by a station close to the experimental greenhouses in El Raal, Murcia, Spain.

		27 Feb	9 Mar	17 Mar	23 Mar	27 Mar	6 Apr	20 Apr
Temperature (°C)	Average	12.90	16.37	15.06	12.25	11.15	12.09	14.24
	Maximum	17.20	23.63	22.42	16.86	14.85	18.09	20.29
	Minimum	8.70	9.44	8.36	7.64	8.23	6.89	8.88
Relative Humidity (%)	Average	75.47	50.65	69.01	84.53	85.91	82.01	81.37
Hours of Sunshine/Day	Average	8.44	9.50	8.67	7.14	5.80	9.09	9.47

## Data Availability

Data have been addressed in the manuscript and in the Supplementary Material.

## Supplementary Materials

The following are available online. Figure S1: Biometrical characteristics of phenological stages in ‘Lamuyo’ green pepper fruit: weight (g), length (mm), and diameter (mm). Data are the mean ± SE.

## References

[1] Cisternas-Jamet J., Salvatierra-Martínez R., Vega-Gálvez A., Stoll A., Uribe E., Goñi M.G. (2020). Biochemical composition as a function of fruit maturity stage of bell pepper (*Capsicum annum*) inoculated with Bacillus amyloliquefaciens. Sci. Hortic..

[2] Blanco-Ríos A.K., Medina-Juárez L.A., González-Aguilar G.A., Gámez-Meza N. (2013). Antioxidant activity of the phenolic and oily fractions of different sweet peppers. J. Mex. Chem. Soc..

[3] Cortés-Estrada C.E., Gallardo-Velázquez T., Osorio-Revilla G., Castañeda-Pérez E., Meza-Márquez O.G., López-Cortez M.S., Hernández-Martínez D.M. (2020). Prediction of total phenolics, ascorbic acid, antioxidant capacities, and total soluble solids of *Capsicum annuum* L. (bell pepper) juice by FT-MIR and multivariate analysis. LWT.

[4] Loizzo M.R., Pugliese A., Bonesi M., Menichini F., Tundis R. (2015). Evaluation of chemical profile and antioxidant activity of twenty cultivars from *Capsicum annuum*, *Capsicum baccatum*, *Capsicum chacoense* and *Capsicum chinense*: A comparison between fresh and processed peppers. LWT Food Sci. Technol..

[5] Alvarez-Parrilla E., de la Rosa L.A., Amarowicz R., Shahidi F. (2011). Antioxidant Activity of Fresh and Processed Jalapeño and Serrano Peppers. J. Agric. Food Chem..

[6] Shotorbani N.Y., Jamei R., Heidari R. (2013). Antioxidant activities of two sweet pepper *Capsicum annuum* L. varieties phenolics extracts and the effects of thermal treatment. Avicenna J. Phytomed..

[7] Howard L.R., Smith R.T., Wagner A.B., Villalon B., Burns E.E. (1994). Provitamin A and ascorbic acid content of fresh pepper cultivars (*Capsicum annuum*) and processed Jalapeños. J. Food Sci..

[8] Valero D., Zapata P.J., Martínez-Romero D., Guillén F., Castillo S., Serrano M. (2014). Pre-harvest treatments of pepper plants with nitrophenolates increase crop yield and enhance nutritive and bioactive compounds in fruits at harvest and during storage. Food Sci. Technol. Int..

[9] Manikharda M., Takahashi M., Arakaki M., Yonamine K., Hashimoto F., Takara K., Wada K. (2018). Influence of fruit ripening on color, organic acid contents, capsaicinoids, aroma compounds and antioxidant capacity of Shimatogarashi (*Capsicum frutescenes*). J. Oleo Sci..

[10] Hamed M., Kalita D., Bartolo M.E., Jayanty S.S. (2019). Capsaicinoids, Polyphenols and Antioxidant Activities of *Capsicum annuum*: Comparative Study of the Effect of Ripening Stage and Cooking Methods. Antioxidants.

[11] Chávez-Mendoza C., Sanchez E., Muñoz-Marquez E., Sida-Arreola J.P., Flores-Cordova M.A. (2015). Bioactive Compounds and Antioxidant Activity in Different Grafted Varieties of Bell Pepper. Antioxidants.

[12] Ghasemnezhad M., Sherafati M., Payvast G.A. (2011). Variation in phenolic compounds, ascorbic acid and antioxidant activity of five coloured bell pepper *(Capsicum annum)* fruits at two different harvest times. J. Funct. Foods.

[13] Martí M.C., Camejo D., Vallejo F., Romojaro F., Bacarizo S., Palma J.M., Sevilla F., Jiménez A. (2011). Influence of fruit ripening stage and harvest period on the antioxidant content of sweet pepper cultivars. Plant Food Hum. Nutr..

[14] Howard L.R., Talcott S.T., Brenes C.H., Villalon B. (2000). Changes in phytochemical and antioxidant activity of selected pepper cultivars (*Capsicum species*) as influenced by maturity. J. Agric. Food Chem..

[15] Nováková L., Solich P., Solichová D. (2008). HPLC methods for simultaneous determination of ascorbic and dehydroascorbic acids. Trends Anal. Chem..

[16] Zhuang Y., Chen L., Sun L., Cao J. (2012). Bioactive characteristics and antioxidant activities of nine peppers. J. Funct. Foods.

[17] Yahia E.M., Contreras-Padilla M., Gonzalez-Aguilar G. (2001). Ascorbic acid content in relation to ascorbic acid oxidase activity and polyamine content in tomato and bell pepper fruits during development, maturation and senescence. Lebensm. Wiss. Technol..

[18] Carr A.C., Frei B. (1999). Toward a new recommended dietary allowance for vitamin C based on antioxidant and health effects in humans. Am. J. Clin. Nutr..

[19] Navarro J.M., Flores P., Garrido C., Martinez V. (2006). Changes in the contents of antioxidant compounds in pepper fruits at different ripening stages, as affected by salinity. Food Chem..

[20] Márkus F., Daood H.G., Kapitany J., Biacs P.A. (1999). Change in the carotenoid and antioxidant content of spice red pepper (paprika) as a function of ripening and some technological factors. J. Agric. Food Chem..

[21] Marín A., Ferreres F., Tomás-Barberán F., Gil M.I. (2004). Characterization and quantification of antioxidant constituents of sweet pepper. J. Agric. Food Chem..

[22] Mozafar A. (1994). Plant Vitamins: Agronomic, Physiological and Nutritional Aspects.

[23] Martí M.C., Camejo D., Olmos E., Sandalio L.M., Fernández-García N., Jiménez A., Sevilla F. (2009). Characterization and changes in the antioxidant system of chloroplasts and chromoplasts isolated from green and mature pepper fruits. Plant Biol..

[24] Jiménez A., Creissen G., Kular B., Firmin J., Robinson S., Verhoeyen M., Mullineaux P. (2002). Changes in oxidative processes and components of the antioxidant system during tomato fruit ripening. Planta.

[25] Lee S.K., Kader A.A. (2000). Preharvest and postharvest factors influencing vitamin C content of horticultural crops. Postharvest Biol. Technol..

[26] Serna-Escolano V., Valverde J.M., García-Pastor M.E., Valero D., Castillo S., Guillén F., Martínez-Romero D., Zapata P.J., Serrano M. (2019). Pre-harvest methyl jasmonate treatments increase antioxidant systems in lemon fruit without affecting yield or other fruit quality parameters. J. Sci. Food Agric..

[27] Belwal T., Pandey A., Bhatt I.D., Rawal R.S., Luo Z.S. (2019). Trends of polyphenolics and anthocyanins accumulation along ripening stages of wild edible fruits of Indian Himalayan region. Sci. Rep..

[28] Singh R., Rastogi S., Dwivedi U.N. (2010). Phenylpropanoid Metabolism in Ripening Fruits. Compr. Rev. Food Sci. Food Saf..

[29] Pérez-Gálvez A., Viera I., Roca M. (2020). Carotenoids and Chlorophylls as Antioxidants. Antioxidants.

[30] Ribeiro B., Rangel J., Valentão P.C., Andrade P.B., Pereira J.A., Bölke H., Seabra R.M. (2007). Organic acids in two Portuguese chestnut (*Castanea sativa Miller*) varieties. Food Chem..

[31] Barzegar T., Fateh M., Razavi F. (2018). Enhancement of postharvest sensory quality and antioxidant capacity of sweet pepper fruits by foliar applying calcium lactate and ascorbic acid. Sci. Hortic..

[32] Maccarrone M., D′Andrea G., Salucci M.L., Avigliano L., Finazzi-Agrò A. (1993). Temperature, pH and UV irradiation effects on ascorbate oxidase. Phytochemistry.

[33] Tiwari U., Cummins E. (2013). Factors influencing levels of phytochemicals in selected fruit and vegetables during pre- and post-harvest food processing operations. Food Res. Int..

[34] Barbagallo R.N., Chisari M., Patané C. (2012). Polyphenol oxidase, total phenolics and ascorbic acid changes during storage of minimally processed ‘California Wonder’ and ‘Quadrato d’Asti’ sweet peppers. LWT.

[35] Raffo A., Baiamonte I., Paoletti F. (2008). Changes in antioxidants and taste-related compounds content during cold storage of fresh-cut red sweet peppers. Eur. Food Res. Technol..

[36] Mashabela M.N., Selahle K.M., Soundy P., Crosby K.M., Sivakumar D. (2015). Bioactive Compounds and Fruit Quality of Green Sweet Pepper Grown under Different Colored Shade Netting during Postharvest Storage. J. Food Sci..

[37] Serrano M., Zapata P.J., Castillo S., Guillén F., Martinez-Romero D., Valero D. (2010). Antioxidant and nutritive constituents during sweet pepper development and ripening are enhanced by nitrophenolate treatments. Food Chem..

[38] Hernández-López G., Ventura-Aguilar R.I., Correa-Pacheco Z.N., Bautista-Baños S., Barrera-Necha L.L. (2020). Nanostructured chitosan edible coating loaded with α-pinene for the preservation of the postharvest quality of *Capsicum annuum* L. and Alternaria alternata control. Int. J. Biol. Macromol..

[39] Samira A., Woldetsadik K., Workneh T.S. (2013). Postharvest quality and shelf life of some hot pepper varieties. J. Food Sci. Technol..

[40] Castro J.M., Avila V.C.M., Rocha F.M., Ochoa M.A., Gallegos I.A. Effect of controlled atmospheres on quality of green pepper poblano (ancho). Proceedings of the 16th International Pepper Conference.

[41] Medlicott A.P., Thompson A.K. (1985). Analysis of sugars and organic acids in ripening fruits by high performance liquid chromatography. J. Sci. Food Agric..

[42] Mizrach A., Filtsanov U., Fuchs V. (1997). An ultrasonic nondestructive method for measuring maturity of fruit. Trans Asae.

[43] Peña-Estévez M.E., Artés-Hernández F., Artés F., Aguayo E., Martínez-Hernández G.B., Galindo A., Gómez P.A. (2016). Quality changes of pomegranate arils throughout shelf life affected by deficit irrigation and pre-processing storage. Food Chem..

[44] Sayyari M., Babalar M., Kalantari S., Martínez-Romero D., Guillén F., Serrano M., Valero D. (2011). Vapour treatments with methyl salicylate or methyl jasmonate alleviated chilling injury and enhanced antioxidant potential during postharvest storage of pomegranates. Food Chem..

[45] García-Pastor M.E., Zapata P.J., Castillo S., Martínez-Romero D., Guillén F., Valero D., Serrano M. (2020). The Effects of Salicylic Acid and Its Derivatives on Increasing Pomegranate Fruit Quality and Bioactive Compounds at Harvest and during Storage. Front. Plant Sci..

