# Supplementary material for: Influence of the Phenological Stage and Harvest Date on the Bioactive Compounds Content of Green Pepper Fruit
Source: Molecules. 2021 May 22;26(11):3099. doi: 10.3390/molecules26113099 (PMC8196862; doi:10.3390/molecules26113099)
Supplement: Supplementary file 1 [file molecules-26-03099-s001.zip › molecules-1209758-supplementary.pdf]

Influence of the phenological stage and harvest date on the bioactive compounds content of green pepper fruit

Alicia Dobón-Suárez [1], María J. Giménez [1], Salvador Castillo [1], María E. García-Pastor [1] and Pedro J. Zapata [1,*]

[1] Department of Food Technology, EPSO, University Miguel Hernández, Ctra. Beniel km. 3.2, 03312 Alicante, Spain; alicia.dobon@goumh.umh.es (A.D.-S.); maria.gimenezt@umh.es (M.J.G.); scastillo@umh.es (S.C.); m.garciap@umh.es (M.E.G.-P.)

* Correspondence: pedrojzapata@umh.es; Tel.: +34-966749789

Supplementary Material

Figure S1. Biometrical characteristics of phenological stages in 'Lamuyo' green pepper fruit: weight (g), length (mm), and diameter (mm). Data are the mean ± SE.

'Lamuyo' Phenological Stages

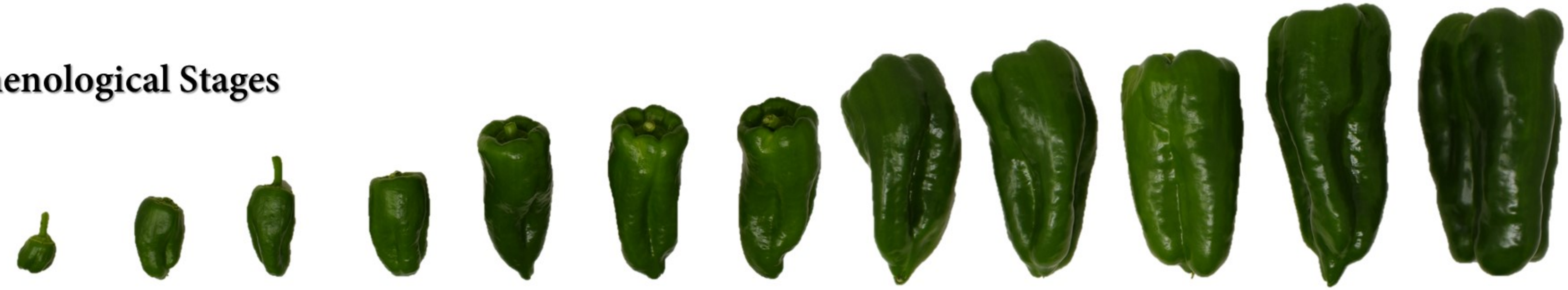

	S1	S2	S3	S4	S5	S6	S7	S8	S9	S10	S11	S12
Weight (g)	5.30±0.21	18.12±0.45	18.14±0.47	37.08±0.97	72.75±1.97	82.12±2.57	95.23±3.47	103.20±4.02	130.91±4.52	158.01±4.92	181.14±5.12	252.68±5.78
Lenght (mm)	23.31±0.78	48.35±1.08	52.97±1.25	60.01±1.80	84.32±2.80	91.78±3.10	96.25±3.01	117.44±4.19	118.47±4.04	114.97±4.27	143.01±4.74	135.74±4.22
Diameter (mm)	23.23±0.87	31.64±1.22	30.52±1.44	37.62±1.30	44.86±1.04	50.22±1.40	52.87±1.72	62.58±1.42	65.70±1.56	64.22±1.63	66.58±1.87	75.40±1.99
